# Involvement of CXCR4 Chemokine Receptor in Metastastic HER2-Positive Esophageal Cancer

**DOI:** 10.1371/journal.pone.0047287

**Published:** 2012-10-17

**Authors:** Stephanie J. Gros, Nina Kurschat, Astrid Drenckhan, Thorsten Dohrmann, Evelyn Forberich, Katharina Effenberger, Uta Reichelt, Robert M. Hoffman, Klaus Pantel, Jussuf T. Kaifi, Jakob R. Izbicki

**Affiliations:** 1 Department of General, Visceral, and Thoracic Surgery, University Medical Center Hamburg-Eppendorf, Hamburg, Germany; 2 Department of Diagnostic and Interventional Radiology, University Medical Center Hamburg-Eppendorf, Hamburg, Germany; 3 Department of Tumor Biology, University Medical Center Hamburg-Eppendorf, Hamburg, Germany; 4 Department of Pathology, Charite, Campus Benjamin Franklin, Berlin, Germany; 5 AntiCancer, Inc., San Diego, California, United States of America; 6 Department of Surgery, University of California San Diego, San Diego, California, United States of America; University of South Alabama, United States of America

## Abstract

A functional linkage of the structurally unrelated receptors HER2 and CXCR4 has been suggested for breast cancer but has not been evaluated for esophageal carcinoma. The inhibition of HER2 leads to a reduction of primary tumor growth and metastases in an orthotopic model of esophageal carcinoma. The chemokine receptor CXCR4 has been implicated in metastatic dissemination of various tumors and correlates with poor survival in esophageal carcinoma. The aim of this study was to investigate a correlation between the expression levels of HER2 and CXCR4 and to evaluate the involvemnent of CXCR4-expression in HER2-positive esophageal carcinoma. The effects of HER2-inhibition with trastuzumab and of CXCR4-inhibition with AMD3100 on primary tumor growth, metastatic homing, and receptor expression were evaluated *in vitro* and in an orthotopic model of metastatic esophageal carcinoma using MRI for imaging. The clinical relevance of HER2- and CXCR4-expression was examined in esophageal carcinoma patients. A significant correlation of HER2- and CXCR4-expression in primary tumor and metastases exists in the orthotopic model. Trastuzumab and AMD3100 treatment led to a significant reduction of primary tumor growth, metastases and micrometastases. HER2-expression was significantly elevated under AMD3100 treatment in the primary tumor and particularly in the metastases. The positive correlation between HER2- and CXCR4-expression was validated in esophageal cancer patients. The correlation of CXCR4- and HER2-expression and the elevation of HER2-expression and reduction of metastases through CXCR4-inhibition suggest a possible functional linkage and a role in tumor dissemination in HER2-positive esophageal carcinoma.

## Introduction

The human epithelial growth factor receptor family (HER, ErbB) of receptor kinases plays an important role in tumor growth and progression [1]. Among these receptors, HER2 is the strongest oncogene and is found to be amplified and overexpressed in about 20% of breast cancers [2] In breast cancer HER2 is known to be associated with poor prognosis and metastases [3], [4]. HER2-overexpression and amplification is reported in esophageal cancer with a tendency towards higher rates of positivity in adenocarcinoma [5]–[10] compared to squamous cell carcinomas [6], [7], [11]–[13]

A strong concordance of the HER2 status in primary and metastatic esophageal adenocarcinoma with high-level HER2 gene amplification as been observed, suggesting esophageal cancer patients with HER2-positive primary tumors as candidates for trastuzumab therapy [14]. HER2 is known to increase the metastastic potential in murine and human cancer cell lines [15]–[18].

With trastuzumab (Herceptin®) an antibody-based therapy exists which is successfully used clinically for targeting HER2 in metastatic HER2-positive breast cancer [3], [19]–[21]. Trastuzumab application also has a dramatic effect in HER2-positive breast cancer patients as adjuvant therapy [22]. There is even evidence for a possible response of HER2-positive non-breast cancers (e.g. gastric cancer) to trastuzumab [23], [24].

A significant reduction of primary tumor growth and of metastatic spread has previously been reported in an orthotopic model of HER2-positive esophageal adenocarcinoma under treatment with trastuzumab [25]. It has been shown that HER2- signalling in breast cancer enhances the expression of CXCR4, which is required for HER2-mediated invasion [26]. The chemokine CXCR4 has been suggested to play an essential role in tumor cell homing to lymph nodes and bone marrow in esophageal carcinoma [27]. Expression of CXCR4 correlates significantly with overall and tumor-specific survival in esophageal carcinoma and is associated with poor prognosis [27]. A correlation of CXCR4 and HER2 and possible functional role in the interaction of their pathways, however, has not been investigated for adenocarcinoma of the esophagus.

Chemokines are a superfamily of small cytokine-like peptides [28], [29]. Through interaction with the chemokine receptors, chemokines induce cytoskeletal rearrangement of hematopoietic cells, increase their adhesion, and direct migration to home-specific organs. Chemokine receptors are G-protein-coupled receptors and CXCR4 is one of the best characterized receptors. CXCR4 and its ligand SDF-1α play an important role in targeting breast cancer metastases [30], [31]. The chemokine SDF-1α is released in high amounts by organs such as lung, bone, and liver. The attraction of SDF-1α and CXCR4 causes breast cancer cells to migrate into these organs, where they proliferate and form metastastes [30], [31].

As metastasis is still the leading cause of tumor-related death and morbidity it is essential to further understand the complex pathophysiologic pathways and processes leading to metastatic spread. Metastatic pathways of malignant tumor disease are complex and still poorly understood [32]–[36].

The aim of this study was to investigate the effects of single and combined inhibition of HER2 and CXCR4 receptor pathways, and to examine HER2- and CXCR4-expression levels under inhibition in order to determine a possible involvement of CXCR4-expression in HER2-positive esophageal carcinoma. Moreover, the aim was to further investigate the role of CXCR4 and HER2 in primary tumor growth and in the homing of metastases. Besides determining the importance of the presence of HER2 and CXCR4 in a representative patient collective, a highly metastatic model of esophageal carcinoma was used for evaluation.

## Materials and Methods

### Cell Line, cell proliferation, and cell migration *in vitro*

The human cell line OE19 (European Collection of Cell Cultures (ECACC), Health Protection Agency, Wiltshire, UK) was cultured in RPMI1560 medium (Biochrome KG, Berlin, Germany) as previously described [25].

Cell proliferation was measured using the LDH Cytotoxicity Kit (PromoKine, Heidelberg, Germany). 50000 OE19 cells were seeded into a 24-well plate and grown overnight. AMD3100 (Sigma-Aldrich, Munich, Germany) was supplemented to the culture medium and cell vitality was analysed after 48 hours.

Tumour cell migration through a microporous membrane was assessed based on the Boyden chamber principle. Cells were incubated with culture medium for 90 min, and then plated onto the top chamber. Culture medium containing 500 ng/ml of recombinant human SDF-1α (R&D Systems, Mineapolis, USA) was added into the lower chamber. The plate was incubated at 37°C, 5%CO_2_ for 18 hrs. The migrated cells were stained using DAPI (Sigma-Aldrich, Munich, Germany) and counted under a fluorescence microscope (Carl Zeiss, Jena, Germany).

### Tumor model and therapeutic treatment

NMRI/nu (U.S. Naval Medical Research Institute) mice were obtained from Charles River Deutschland (Sulzfeld, Germany) at 10 weeks of age. All animal procedures were performed in accordance with a protocol approved by the Behörde für Wissenschaft und Gesundheit (Freie und Hansestadt Hamburg, Germany). The esophageal carcinoma implantation model was obtained as previously described [25], [37], [38]. Mice were weighed and examined for tumor development every other day.

After primary tumor growth was established by magnetic-resonance-imaging (MRI) on day 14, mice were randomised into four groups of nine mice each (ten mice in the control group). Group one was treated biweekly with an intraperitoneal injection of 20 mg/kg body weight trastuzumab (Roche, Penzberg, Germany) in a volume of 100 µl. Group two received 5 mg/kg body weight AMD3100 (Sigma-Aldrich, Munich, Germany) in 100 µl by intraperitoneal injection. Group three received both daily AMD3100 injections as well as biweekly trastuzumab. Group was for given daily intraperitoneal sham injections with 100 µl PBS and used as a control group.

### Immunohistochemistry

The HercepTest (DAKO, Glostrup, Denmark) was used according to the protocol of the manufacturer, using a 1∶300 dilution of the primary antibody. CXCR4-Staining was performed using the primary rabbit polyclonal CXCR4 antibody (Abcam, clone 2074, Cambridge, UK) at a dilution of 1∶250 overnight at 4°C. The antibody reaction was developed with the Cell and Tissue Staining Kit, using the HRP-AEC-System, from R&D-Systems (Minneapolis, MN, USA). Sections were counterstained with Mayer's hematoxylin solution (Merck). Tumor tissue was identified by hematoxylin eosin (HE) staining. Immunostaining was scored by one pathologist (U.R.) and a second independent examiner (A.D.).following a four-step scale (0,1þ,2þ,3þ) according to the manufacturer's directions

### Fluorescence in situ hybridization (FISH)

*HER2* FISH analysis was performed using the *HER2*FISH pharmDx™ Kit (DAKO, Glostrup, Denmark) according to the manufacturers protocol.

### Detection of micrometastases

Total RNA was isolated from liver and lung samples with an RNA isolation kit (Qiagen, Hilden, Germany) and reverse transcribed with a high-capacity cDNA reverse-transcription kit (Applied Biosystems). Micrometastases were detected by mRNA expression of the human *gapdh* gene by real-time PCR analysis. Results were normalised using *18S* RNA expression of the tissue samples. PCR primers (TaqMan Gene Expression Assay Gapdh human Hs99999905_m1, Partnumber 4351370, TaqMan Gene Expression Assay 18S Hs99999901_s1) and TaqMan Universal PCR Mastermix were obtained (Applied Biosystems). Micrometastases data are presented as delta-ct-values.

### Detection of disseminated tumor cells in bone marrow

Bone marrow was sampled from the femur of mice at the time of sarifice and isolated by density gradient as previously described [39]. Slides with bone marrow cells were immunocytochemically assessed for disseminated tumor cells using the monoclonal antihuman anticytokeratin antibody AE1/AE3 (Dako, Glostrup, Denmark) labeled with fluorochrome FITC and anti-HER2 monoclonal antibody NCL-CB11 (Novocastra Reagents and Antibodies, Leica Microsystems, Wetzlar, Germany) according to the manufacturers protocols. After staining, slides were covered with Vectashield Mounting Medium containing Dapi (Vector Laboratories, Burlingame, CA).

### Magnetic resonance imaging

All MRI measurements were performed on a clinical whole-body 3T MRI scanner (Intera®, Philips, Netherlands) using a dedicated small animal solenoid coil (PFL-HH, Hamburg, Germany). Mice were anaesthetised as previously described [25], [37]. The MRI was equipped with a standard gradient system with a max. amplitude of 40 mTm^−1^ and a slew rate of 150 Tm^−1^ s^−1^. After a short survey, coronal T1 and T2 as well as sagittal T2–weighted sequences were used for tumor visualization. First, coronal T1 and T2 turbo spin echo sequences (TSE) were conducted. Imaging parameters for coronal sequences: T1 weighted TSE:repetition time (TR) = 1275 msec, echo time (TE) = 33 msec, flip angle = 90°, number of slices = 14, slice thickness = 1 mm, matrix = 464×480 px, FOV = 100 mm, number of excitations (NEX) = 3, reconstructed voxel = 0.21/0.21/1 mm^3^, echo train length (ETL) = 4; coronal T2–weighted TSE with fat saturation: TR = shortest; TE = 90 msec, flip angle = 90°, number of slices = 20, slice thickness = 1 mm, matrix = 448×448 px, FOV = 100 mm, number of excitations = 3 and reconstructed voxel = 0.22/0.22/1 mm^3^. Thereafter, a sagittal T2 TSE sequence using fat saturation was acquired according to the following parameters: TR = shortest; TE = 90 msec, flip angle = 90, number of slices = 22, slice thickness = 1 mm, matrix = 448×448 px, FOV = 100 mm, NEX = 3 and reconstructed voxel = 0.22/0.22/1 mm^3^. Examination time was ∼17 min.

### Image analysis and volumetric measurement

DICOM images were processed using the free available software Osirix®. The largest tumour diameter was measured in the sequences which best visualized the tumour. Measurements were performed separately by two researchers for each mouse before and after therapy. Furthermore, the tumour volume was obtained by manual circling of the tumour rim on each slice, followed by the automatic construction of a 3D tumour map.

### Patient collective, immunostaining of human tissue and data analysis

A patient collective of 202 patients, that underwent surgery in curative intention in the Department of Surgery at the University Medical Center Hamburg-Eppendorf, was examined for both HER2- and CXCR4-expression by immunostaining. This study was approved by the ethics committee of the chamber of physicians at Hamburg, Germany and written consent was obtained from all patients to use the resected samples. Upon histopathological examination the resection margins were tumor free. Tumor stage and grade were classified according to the tumor-node metastasis classification of the International Union Against Cancer [40]. Tumor tissue samples that had been snap-frozen in liquid nitrogen were embedded in Optimal Cutting Temperature Compound (Tissue-Tek) and sectioned at 5 mm. For HER2 staining the HercepTest (DAKO, Glostrup, Denmark) was used according to the protocol of the manufacturer, using a 1∶300 dilution of the primary antibody. Immunostaining was scored by the pathologist (U.R.), following a four-step scale (0,1þ,2þ,3þ) according to the manufacturer's directions. Sections were stained with anti-human CXCR4 monoclonal antibody (IgG2a, clone12G5; R&D Systems, Minneapolis, MN) at a dilution of 1∶100 overnight at 4°C. An irrelevant murine IgG1 monoclonal antibody (MOPC21; Sigma, Deisenhofen, Germany) was used as a negative control. The antibody reaction was developed with the alkaline phosphatase anti-alkaline phosphatase (APAAP) technique including secondary rabbit anti-mouse polyclonal antibody (clone Z0259; Dako, Hamburg, Germany) and APAAP staining complex (Dako) combined with a new fuchsin stain (Sena, Heidelberg, Germany). For visualization, sections were counterstained with Mayer's hematoxylin solution (Merck). Specimens were considered immunopositive for CXCR4 when more than 20% of all tumor cells within one section were clearly immunostained. Results did not vary when another cutpoint (e.g.5% positive tumor cells as a cutpoint) was tested. The tumor samples were then classified as having either absent to low staining (CXCR4-negative) if 20% or fewer tumor cells expressed CXCR4, or moderate to strong staining (CXCR4-positive) if more than 20% of tumor cells expressed CXCR4. Immunohistochemical analysis and scoring were performed by two independent investigators (U.R.,S.G.).

Statistical analysis was performed on a standard personal computer using SPSS for Windows (version 11.5.1; SPSS Inc., Chicago, IL). Correlations were calculated with cross-tables and statistical significance was determined by Fisher's test with a p-value from two-sided tests of <.05.

### Statistical data analysis

Statistical data analysis of *in vivo* data was performed using PASW Statistics 18 (SPSS Inc., Chicago, USA) on a standard personal computer with a quadcore processor. For determination of significance concerning differences in tumor growth and receptor expression, the non-parametric Mann–Whitney-Wilcoxon-Test was used. Fisher's-Exact-Test for Count-Data was used for determination of significance of metastases. For correlation of MRI tumor volume and tumor weight the Pearson-coefficient and for correlation of HER2- and CXCR4-expression the Spearman's-rank-correlation-coefficient was used.

## Results

The aim of this study was investigate a possible interaction of the HER2- and CXCR4-receptors and their expression levels under treatment with their respective inhibitors in order to determine an impact of CXCR4-expression in HER2-positive esophageal carcinoma.

### *In vitro* proliferation and migration of esophageal OE19 cells

To investigate the effects of the CXCR4- and HER2-receptors on cell proliferation *in vitro* OE19 cells were treated with AMD3100 and trastuzumab, respectively. While it was expected that trastuzumab treatment leads to a cell growth reduction, the effect of treatment with AMD3100 had not been defined. Cell migration of the CXCR4-positive cells however should be influenced by the chemoattractant SDF-1α.

**Figure 1:**
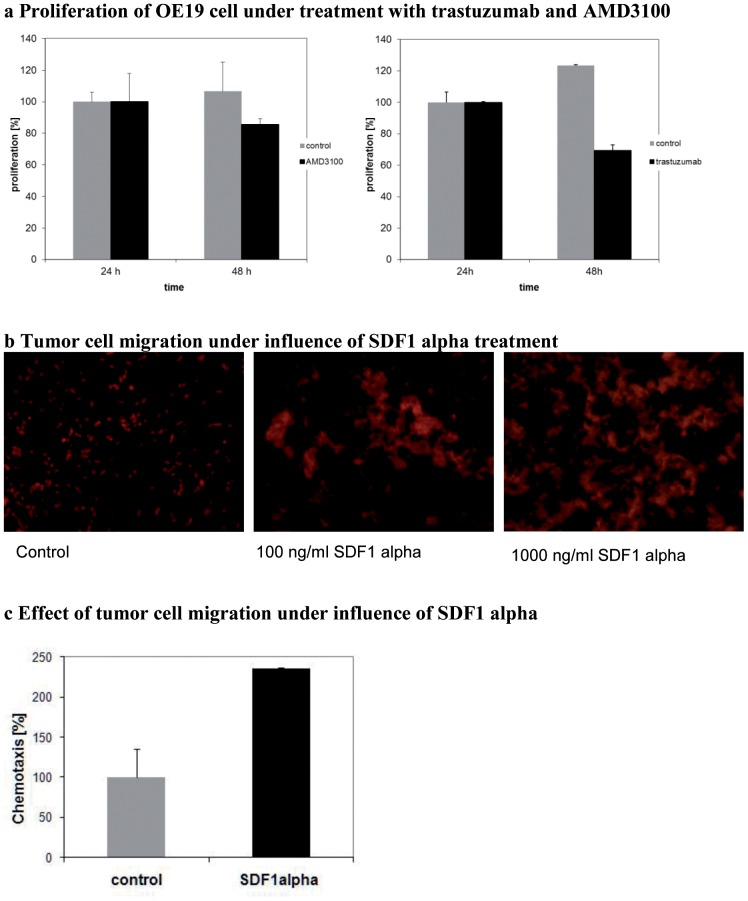
**A** Effect of trastuzumab and ADM3100 treatment on proliferation (%) of OE19 cells compared to control in the lactate-dehydrogenase assay. Receptor inhibition leads to reduced proliferation of cells. It shows a significant reduction of cell proliferation under HER2- and CXCR4-receptor inhibition after treatment with trastuzumab (p = 0.005) as well as with AMD3100 (p = 0.02) compared to the untreated control. **B** Microscopic evaluation shows dose-dependent effect of SDF-1α-stimulated cell migration on OE19 cells. **C** A relevant effect of SDF-1α on cell migration is observed at 250 ng/ml compared to unstimulated cells (control).

The *in vitro* cell proliferation assays reproducibly showed a significant reduction of cell proliferation under HER2- and CXCR4-receptor inhibition after treatment with trastuzumab (p = 0.005) as well as with AMD3100 (p = 0.02) (Figure 1A). As expected, treatment with trastuzumab led to a stronger reduction of proliferation than treatment with AMD3100. In the migration assay a dose-dependent effect on cell migration could be observed after treatment with SDF-1α (Figure 1B). Chemotaxis of CXCR4-positive OE19 cells could thus be induced by SDF-1α (Figure 1C). Results were reproduced with several concentrations (data not shown).

### *In vivo* proliferation of primary tumor in the orthotopic model

**Figure 2:**
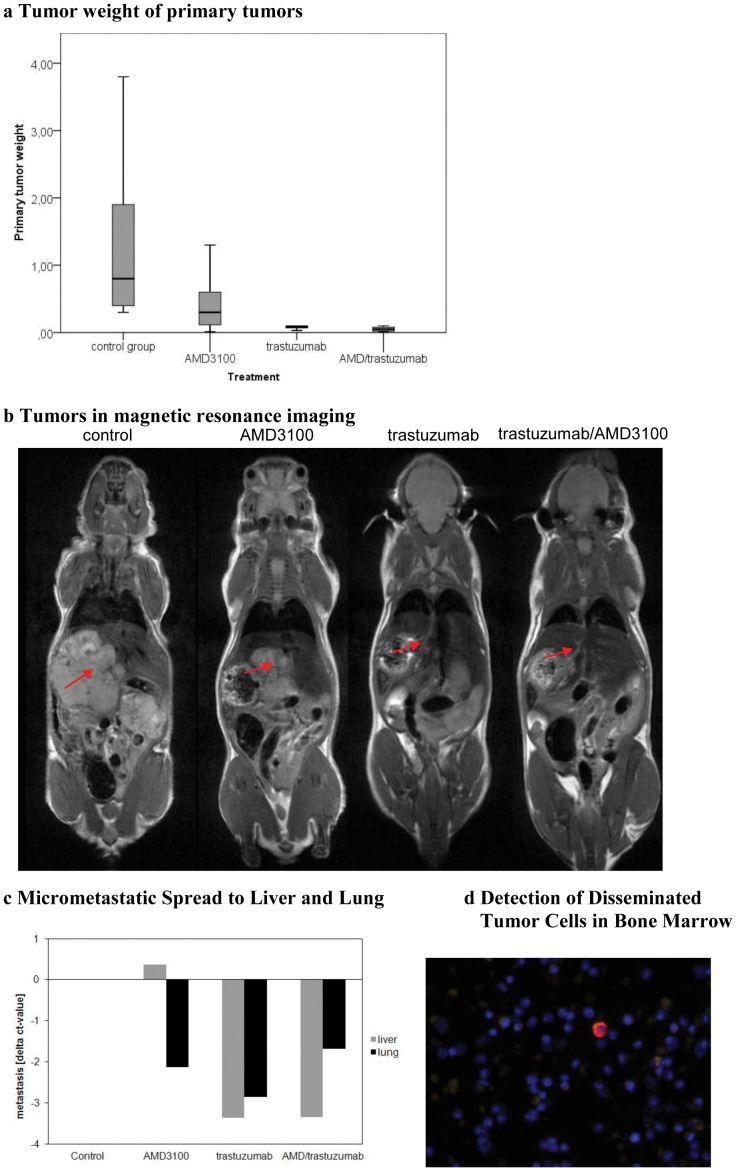
**A** Significant differences in tumor weights between control and trastuzumab-treated groups (p<0.00), control and combination trastuzumab/AMD3100-treated groups (p<0.00), trastuzumab and AMD3100-treated groups (p = 0.04), and AMD and combination trastuzumab/AMD3100-treated groups (p = 0.02). Although the effect of AMD3100 on the primary tumor weight was not as relevant as the effect of trastuzumab, a potent effect was achieved by AMD3100 treatment alone, compared to the untreated group. **B** MRI-based tumor volumetry confirmed the results of tumor weight. The tumor weights at time of autopsy correlated significantly with the volumetric measure by MRI (correlation coefficient: 0.837, p<0.01). **C** Micrometastases in liver and lung after treatment with AMD3100 and trastuzumab, were analysed by real-time PCR according to the level of human *gapdh*. AMD3100 and trastuzumab-treated mice showed with a mean delta-ct-value of −2 and −3 strong reductions in lung metastasis of 75 to nearly 100%. Additionally the trastuzumab-treated mice had a strong reduction in liver metastasis represented by a mean delta-ct-value of −3. The AMD3100/trastuzumab combination group had a reduced rate of lung (delta-ct −2), and liver (delta-ct −3) metastasis. **D** Disseminated tumor cells were detected by cytokeratin and HER2 immunhistochemical staining. Figure 3B shows a bone marrow sample. Human cell with a strong positivity for HER2 is detectable (red). * Due to space limitations, AMD3100 was abbreviated to AMD in *Figures 2a and c* *.*

To further investigate the behaviour of local and metastatic esophageal tumor growth in *vivo* OE19 cells were implanted orthotopically into NMRI/nu mice. All mice developed primary tumors at the implantation site as shown by MRI two weeks after implantation. The bodyweight of the mice ranged from 18.5–26.3 g at the beginning and 22.5–32.7 g at the termination of the experiment. The means of bodyweights of each therapeutic group at the beginning and termination of the experiment are summarized in Table 1. No significant variation was observed. Mice were randomized two weeks after implantation into therapeutic groups. We have previously shown that tumor sizes two weeks after implantation were comparable between groups [37]. At the time of termination of the experiment, an MRI scan was performed immediately before dissecting the animals. All animals reached the end point of the study without severe weight loss or other signs of tumor disease. Tumor weights were recorded and gave values between 0.01–3.9 g. Tumor volumetry was performed and confirmed the tumor weight results (Table 1). While weight values within the control, trastuzumab-treated group, and trastuzumab/AMD3100-treated group were more homogenous, values varied more strongly within the AMD3100-treated group. Tumor weights in the control group were significantly higher than in the trastuzumab-treated (p<0.0001) and trastuzumab/AMD3100-treated (p<0.0001) groups. Tumor weights in the AMD3100-treated group were significantly higher than in the trastuzumab-treated (p = 0.04) and trastuzumab/AMD3100-treated (p = 0.02) groups (Figure 2A). Although the effect of AMD3100 on the primary tumor weight was not as significant as the effect of trastuzumab, a potent effect was achieved by AMD3100 treatment alone compared to the untreated group. The tumor weights at time of autopsy correlated significantly with the volume measured by MRI (correlation coefficient: 0.837, p<0.01). Representative examples of magnetic resonance images for tumor evaluation with and without treatment are shown in Figure 2B.

**Table 1:** Mean Body Weight, Tumor Weight and Volume of Mice.

	Mean Body Weight of mice (g)		Tumor Weights (g) [Mean (g)]	Tumor Volume (ml) [Mean (ml)]
	Beginning	Termination		
Control	21.94	27.55	0.3–3.8 [1.4 ]	0.2266–1.3797 [0.620985]
AMD3100	21.811	26.556	0.01–3.9 [0.8]	0.1956–3.3888 [1.1978]

Summary of mean body weights of mice at the beginning of treatment and at the termination of the experiment. No significant differences between treatment groups were seen. Tumor weights and tumor volumes are summarized for each treatment group. A positive correlation of tumor weight and volume was noted (correlation coefficient: 0.837, p<0.01).

### Metastastic potential of esophageal tumor cells *in vivo*

At the termination of the *in vivo* experiment, potentially metastatic tissues (lung, liver and lymph nodes) were sampled and histologically examined to evaluate the metastatic spread of the esophageal tumor for each treatment group. Additionally, micrometastases in liver and lung were detected by mRNA expression of the human *gapdh* gene by real-time PCR analysis from total RNA.

In the control group extensive metastastic spread was observed to lung, liver and lymph nodes. 20% of animals had aggressive metastatic spread to all compartments including lung, liver and multiple lymph nodes. In contrast, the AMD3100-treated group showed fewer metastases and only one animal expressed highly aggressive metastatic spread to three compartments (lung, liver, and lymph nodes). While metastatic spread in the combination-therapy group was not significantly lower than in the trastuzumab-treated group, metastasis presented solitarily.

When examining overall metastasic spread in H.E. and immunohistological staining there was significantly less metastatic spread found in the trastuzumab-treated group compared to the control group (p = 0.002). In particular, a significant reduction of lung metastases could be observed compared to the control group after AMD3100, trastuzumab and combined treatment as well as to the liver after trastuzumab and combined treatment (Figure 2C). Micrometastic values are presented as delta ct-values of the control and treated groups.

### Disseminated tumor cells in bone marrow

To determine the presence of disseminated tumor cells in the bone marrow, disseminated tumor cells (DTC) were isolated from bone marrow of mice by density gradient and visualized by chromagen immunostaining. The finding of cytokeratin-positive DTC in the bone marrow indicated the extent of tumor disease (Figure 2D).

### CXCR4 and HER2 expression profile of OE19 cells *in vivo*

**Figure 3:**
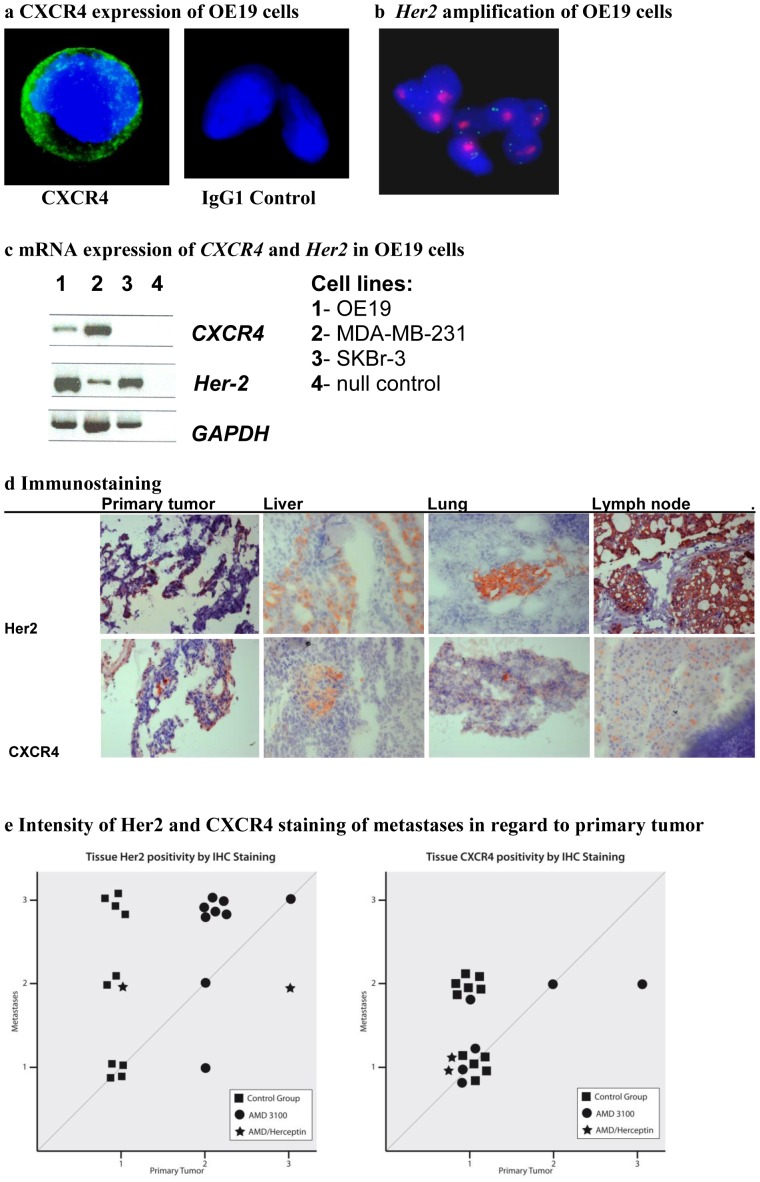
**A** CXCR4-expression of OE19 cells determined by fluorescence immunostaining (IgG1-control) **B** Confirmation of *Her2*-amplification determined by fluorescence in situ hybridization (red: *Her2*-gene loci, green reference *CENT-17*-loci) **C** *CXCR4* and *HER-2* mRNA-expression analysis of esophageal cancer cell line OE19 compared to MDA-MB-231 and SKBr-3 cell lines and null control (nc). **D** CXCR4 and HER2 expression level analysis determined by immunostaining in primary tumor, liver, lung and lymph node. Representative images are shown from the tissues of an untreated animal (magnification ×100). **E** Intensity of HER2- and CXCR4-expression was scored in primary tumor and metastases. Positivity-scores of primary tumor and respective metastases were matched to evaluate the occurrence of and correlation of primary tumor expression and that of its respective metastases between the therapeutic groups. Trastuzumab treatment led to an absence of metastases and thus could not be included. * Due to space limitations, AMD3100 was abbreviated to AMD in *Figure 3e* *.*

Firstly, OE19 cells were examined for their expression of CXCR4 and HER2. Not only the HER2-overexpression but also the amplification of its gene is of clinical relevance, thus *Her2*-amplification status was verified. OE19 cells showed a strong expression of CXCR4- and HER2-receptors in immunostaining (Figure 3A) as well as an amplification of the *Her2*-gene in FISH (Figure 3B). Semiquantitive mRNA analysis showed expression of *CXCR4* and *Her2* compared with MDA-MB-231 and SKBr-3 cell lines (Figure 3C).

### Correlation of CXCR4- and HER2-expression in the orthotopic *in vivo* model

Secondly, primary tumor and metastatic tissues from the orthotopic model were examined for CXCR4- and HER2-expression. CXCR4- and HER2-expression was observed in all tumor bearing-tissues, including primary tumor, liver, lung and lymph node metastases (Figure 3D). HER2-expression correlated significantly with CXCR4-expression (correlation efficient 0.490, p<0.01).

### Higher intensity of HER2-expression in metastases compared to primary tumor

To further evaluate the relevance of HER2- and CXCR4-correlation, a point-by-point diagram was designed (Figure 3E), in which each metastatic case was marked, indicating both the intensity of expression of the metastasis (y-axis) and the intensity of expression of its respective primary tumor (x-axis). According to the treatment group different symbols were used. The first diagram displays the HER2-intensity, the second the CXCR4-intensity.

Interestingly, a higher expression of HER2 and CXCR4 could be seen in metastases of all therapeutic groups compared to their respective primary tumors. The intensity of HER2- expression (score 1–3) of primary tumors and their respective metastases were applied in the first diagram in Figure 3E. The graph showed that the intensity of the HER2-positivity by immunostaining varies between tissues of treatment groups. While the HER2-positivity of primary tumor in the control group was limited to lighter intensity (score 1), metastases expressed HER2 at all intensities. Under AMD3100 treatment, however, HER2 was only expressed at stronger intensities (scores 2+3) in the primary tumor. Metastases in the AMD3100-treated group express HER2 almost exclusively at the highest intensity (score 3).

When applying the intensity scores of CXCR4-positivity (scores 1–3) of the primary tumors and their respective metastases to the second diagram in Figure 3E, the scores in the control group varied between lighter and medium intensities (scores 1+2). Although the intensity of CXCR4-expression levels of the primary tumors in the AMD3100 treated group reached higher expression levels (score 3), the intensity of metastatic CXCR4-expression did only extent to medium levels (score 2).

In both diagrams, the trastuzumab-treated group could not be evaluated in this way as there were no metastases. For the combined therapy group, there were only two metastatic cases making evaluation statistically not reliable.

### Upregulation of HER2-expression under AMD3100 treatment

Hypothesising that the expression of CXCR4 and/or HER2, their respective pathways and their interaction are involved in mediating tumor progression and metastatic homing, each therapeutic group was evaluated separately as to the intensity of HER2- and CXCR4-expression levels. Using the same diagram (Figure 3E) the intensity of HER2- and CXCR4-expression was compared between the treatment groups. In comparison to the control group, the HER2-intensities of metastases and primary tumors of the AMD3100-treated group are represented by higher scores throughout.

When statistically comparing the HER2 expression of primary tumor and metastases between therapeutic groups, a significantly higher HER2-expression was observed in the trastuzumab-treated group (p = 0.003) and the AMD3100-treated group (p = 0.003) compared to the control group. Upon examination of CXCR4-expression, a significantly higher expression was observed in the trastuzumab-treated group (p = 0.003) and a higher expression in the AMD3100-treated group (p = 0.065) compared to the control group. The combined therapy group (trastuzumab/AMD3100) neither showed significant HER2-expression or CXCR4-expression differences compared to the control group.

### Validation of CXCR4- and HER2-coexpression in human esophageal carcinoma

To further validate the correlation of HER2 and CXCR4 that was found in the *in vivo* studies, primary tumor tissues of 202 patients were examined for both HER2- and CXCR4-expression by immunostaining. Importantly, we have already previously shown that CXCR4-expression in a similar patient collective (136 patients) was associated with poor clinical outcome in esophageal cancer patients [27]. All patients were operated in curative intention. M1 patients with distant lymph node metastases were included. Patient characteristics are summarized in Table 2.

**Table 2:** Patient collective.

Characteristic		Number of patients (%)
**Gender**		
	**Male**	159 (78.7%)
	**Female**	43 (21.3%)
**T-Stage**		
	**T1**	35 (17.3%)
	**T2**	66 (32.7%)
	**T3**	97 (48%)
	**T4**	4 (2%)
**N-Stage**		
	**N0**	75 (37.1%)
	**N1**	127 (62.9%)
**M-Stage**		
	**M0**	147 (86.1%)
	**M1**	28 (13.9%)
**Grading**		
	**G1**	4 (2%)
	**G2**	122 (60.4%)
	**G3**	76 (37.6%)
**Cell Type**		
	**Squamous cell carcinoma**	111 (55%)
	**Adenocarcinoma**	86 (42.6%)
	**Adenoaquamous carcinoma**	5 (2.5%)

Characteristic of 202 patients that were evaluated for CXCR4 and HER2 expression.

Receptor staining was classified into low, medium and high expression. Of the HER2-positive tumor samples, only 14 showed high HER2-expression. In the primary tumor tissue of these patients (n = 14), a significantly positive correlation (p = 0.036) could be observed between high HER2 (57.14%) and high CXCR4 (42.86%) expression (Table 3).

**Table 3:** HER2- and CXCR4-receptor expression.

		CXCR4		Total
		−	+	
HER2	−	154 (81.91%)	34 (18.09%)	188
	+	8 (57.14%)	6 (42.86%)	14
Total		162	40	202

Expression summary of HER2 and CXCR4 in human esophageal carcinoma patients with positive correlation (p = 0.036).

For simplified presentation high receptor expression in this table **is indicated by (+), all other expression levels by (−).**

## Discussion

The orthotopic esophageal carcinoma model in this study mirrors the situation of HER2-positive tumor disease with a positive correlation of HER2- and CXCR4-receptor expression. We show here for the first time that a positive correlation of HER2- and CXCR4-expression exists in esophageal carcinomas of patients. Tumor progression and poor patient prognosis are dependent on a positive CXCR4-receptor status [27].

We show here that OE19 adenocarcinoma cells are susceptible to SDF-1α-mediated migration. Furthermore the extent of tumor disease is stressed by the presence of disseminated tumor cells in the bone marrow.

The inhibition of HER2 with trastuzumab on one hand leads to a significant reduction of primary tumor growth as well as a significant reduction of metastases and to significant changes in the expression levels of HER2 and CXCR4 in the primary tumor. No metastatic spread to liver, lung or lymph nodes is observed under trastuzumab treatment. Inhibition of CXCR4 with AMD3100 on the other hand leads to a significant reduction of primary tumor growth and a relevant reduction of overall metastatic spread and of micrometastatic lung metastases. Although it does not lead to a significant reduction of overall metastases, the metastatic spread presents with solitary metastases and does not appear to be as aggressive as in the control group.

While AMD3100 treatment leads to a significantly higher expression of HER2 in the primary tumor and metastases, metastases exhibit a much higher intensity of HER2 expression than the primary tumor throughout. Although under AMD3100 treatment, CXCR4 expression of the primary tumor is elevated to high levels, CXCR4 expression of metastases matches the expression of the primary tumor and does surpass it. A significantly higher CXCR4 expression is, however, observed in the trastuzumab-treated group and a higher expression in the AMD3100-treated group compared to the control group.

The combined treatment with trastuzumab and AMD3100 leads to a significant reduction of primary tumor growth as well as to a relevant, if not significant reduction of overall metastatic spread and a reduction of micrometastases to liver and lung. This dual-treatment group shows heterogenous levels of HER2 intensity in the only two metastatic cases, CXCR4 is not highly elevated.

### Role of CXCR4 and HER2 in metastatic homing

Cancer metastases result from several highly organized sequential steps involving numerous interactions between the cancer cell and the host, but the detailed molecular mechanisms are still not completely understood [41]. CXCR4 is involved in homing of metastatic spread in numerous tumor entities [30], [31]. In esophageal carcinoma it has been shown that CXCR4 is involved in metastases to lymph nodes and bone marrow and, moreover, is associated with a poor clinical prognosis [27]. The mechanism of CXCR4 upregulation in malignant cells remains poorly understood. CXCR4 was found to be transactivated by hypoxia-induced factor-1α (HIF-1α) at the transcriptional level in renal cell carcinoma [42], [43]. A further study identified enhancement of CXCR4-protein synthesis and inhibition of ligand-induced degradation to be dependent on distant mechanisms of CXCR4-upregulation by HER2 [26]. It has further been suggested that HER2 may inhibit CXCR4-ubiquitination and abrogate subsequent sorting steps and thus prevent degradation [26]. Overall, various possible mechanisms are feasible. The CXCR4-ligand SDF-1α is a small cytokine, secreted locally, is expressed in some tissues, including major homing organs such as lung and bone marrow in breast cancer [31], [44]. The relevance of the presence of disseminated tumor cells in the bone marrow is extensively discussed for various tumors [45]. Its relevance for esophageal carcinoma has been indicated [27] but not yet been determined. Our results suggest an important role of CXCR4, a possible regulation of HER2 though inhibition of the CXCR4 pathway and a subsequent regulation of local tumor progression and metastatic homing of esophageal carcinoma.

### Role of CXCR4- and HER2-receptor expression

While the inhibition of the HER2 receptor through the monoclonal antibody trastuzumab leads to reduction of primary tumor growth as well as significant reduction of metastases to lymph nodes, liver and lung. Inhibition of the CXCR4 receptor reduces metastases compared to the non-treated group in our experiments and leads to an over-expression of HER2 in the primary tumor and even greater expression in the metastases. CXCR4-inhibition also seems to lower CXCR4-expression in metastases compared to the untreated group. The reduction of metastases strengthens the assumption that CXCR4 is indeed involved in metastastic homing in esophageal carcinoma. The combined inhibition of HER2 and CXCR4 leads to further reduction for primary tumor growth. Metastases throughout have a higher HER2-expression level than the primary tumor, suggesting that in metastatic disease HER2 expression is of even greater importance. In an orthotopic model of HER2-positive esophageal carcinoma we have previously shown the significantly positive impact of inhibition of HER2 on primary tumor and on metastases [25].

The present findings support the theory of a strong linkage of two structurally unrelated membrane receptors, HER2 and CXCR4, and thus a functional role of CXCR4 in HER2-positive esophageal carcinoma, which has not been previously described. A similar functional linkage has only been previously established for breast cancer [26]. The group of Li et al. could show that, reciprocally to our findings, HER2 enhances CXCR4 expression, and that increased CXCR4 expression is necessary for HER2-mediated invasion. Furthermore they demonstrated a significant correlation of HER2 and CXCR4 in breast cancer patients, which resulted in a significant worse prognosis. If assuming that HER2 upregulates the expression of CXCR4 [26], this might even suggest that through inhibition of CXCR4, HER2 in rebound is upregulated to again increase CXCR4.

Multiple regulatory mechanisms can be responsible for a functional linkage of the structurally-unrelated receptor cascades of CXCR4 and HER2. It is however known that the parallel activation and transactivation of the ERK/MAPK signalling complex through G-coupled protein receptors, such as CXCR4, and receptor tyrosine kinases, such as HER2, exists. Although the detailed mechanisms of this phenomenon remain unclear, this multi-track signalling is known to lead to proliferation, invasion and migration, finally resulting in tumor progression [46].

Although these coherences have only been previously described for breast cancer [26], our data might further strengthen the theory that HER2-enhanced invasion, migration, adhesion and metastasis is dependent on the upregulation of CXCR4 in esophageal carcinoma. However, further functional analysis will be necessary to investigate the interaction of CXCR4 and HER2 pathways in esophageal carcinoma.

### Conclusion

Overall we show that inhibition of the CXCR4 receptor leads to significant changes in HER2-expression, indicating that blockage of the CXCR4 pathway activates HER2-overexpression. This suggests an involvement of CXCR4 in the HER2-mediated response, which calls for further functional investigation. While the fact that, under inhibition of the HER2 receptor, no metastases occur indicates a reciprocal mechanism, solitary inhibition of CXCR4 still leads to metastases. These coherences have, to our knowledge, not been described for adenocarcinoma of the esophagus before. We could not only confirm the inhibitory effect of trastuzumab treatment on OE19 carcinoma cells in the orthotopic model, but also show a significant tumor growth and reduction of metastases by AMD3100 treatment alone. The positive correlation of high HER2- and CXCR4-expression in the patient collective suggests its relevance not only in the orthotopic animal model, but also its possible scope of application in an assorted patient collective.
