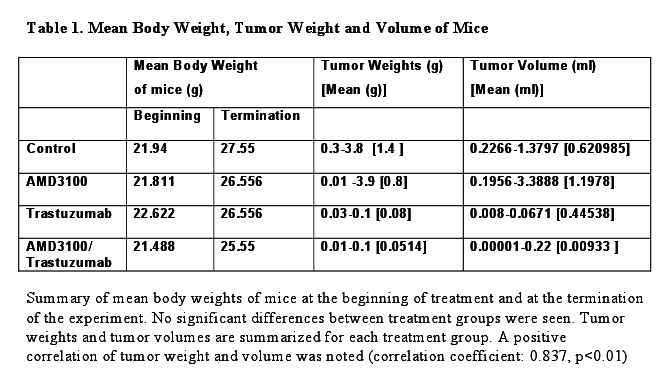# Correction: Involvement of CXCR4 Chemokine Receptor in Metastastic HER2-Positive Esophageal Cancer

**DOI:** 10.1371/annotation/5dafa9a8-2e6d-4487-b35f-7126141fe00e

**Published:** 2013-05-22

**Authors:** Stephanie J. Gros, Nina Kurschat, Astrid Drenckhan, Thorsten Dohrmann, Evelyn Forberich, Katharina Effenberger, Uta Reichelt, Robert M. Hoffman, Klaus Pantel, Jussuf T. Kaifi, Jakob R. Izbicki

The version of Table 1 was incomplete.

The correct, full version of the table is available here: 

**Figure:**